# Detection of foot-and-mouth disease virus rna by reverse transcription loop-mediated isothermal amplification

**DOI:** 10.1186/1743-422X-8-510

**Published:** 2011-11-09

**Authors:** Hao-tai Chen, Jie Zhang, Yong-sheng Liu, Xiang-tao Liu

**Affiliations:** 1 State Key Laboratory of Veterinary Etiologic Biology, National Foot-and-Mouth Disease Reference Laboratory of China, Key laboratory of Animal Virology of Ministry of Agriculture, Lanzhou Veterinary Research Institute, Chinese Academy of Agricultural Sciences, Lanzhou, 730046, Gansu, P.R.China

**Keywords:** Foot-and-mouth disease virus, Detection, Reverse transcription loop-mediated isothermal amplification, Sensitivity, Specificity

## Abstract

A reverse transcription loop-mediated isothermal amplification (RT-LAMP) assay was developed for foot-and-mouth disease virus (FMDV) RNA. The amplification was able to finish in 45 min under isothermal condition at 64°C by employing a set of four primers targeting FMDV 2B. The assay showed higher sensitivity than RT-PCR. No cross reactivity was observed from other RNA viruses including classical swine fever virus, swine vesicular disease, porcine reproductive and respiratory syndrome virus, Japanese encephalitis virus. Furthermore, the assay correctly detected 84 FMDV positive samples but not 65 FMDV negative specimens. The result indicated the potential usefulness of the technique as a simple and rapid procedure for the detection of FMDV infection.

## 1. Background

Foot-and-mouth disease virus (FMDV) is a member of the genus *Aphthovirus *of the family *Picornaviridae*, which is divided into seven serotypes with no cross-protection conferred among the serotypes [1]. Due to the aggressive nature of foot-and-mouth disease (FMD), outbreaks usually result in severe economic losses and impact on both national and international trade within the livestock and animal products [2]. Rapid and accurate diagnosis of any suspected FMD cases is of utmost urgency to control this veterinary infection given the extreme contagiousness of the causative virus.

Conventional laboratory diagnosis of FMD was made by ELISA detection of specific viral antigens and by observation of cytopathic effects in cell culture [3]. Alternatively, the conventional reverse transcriptase polymerase chain reaction (RT-PCR) [4] and real-time RT-PCR [2,5] were developed to complement primary diagnostic techniques for the FMDV infection. These assays were time-consuming and laborious, which required centralized laboratory facilities and clinical specimen submissions, resulted in the delay of FMDV diagnosis. Given these problems, a rapid, simple and practical assay to detect FMDV in animal and its products was therefore required in clinical practice.

A reverse transcription loop-mediated isothermal amplification (RT-LAMP) was applied successfully to the detection of many animal viruses [6-9]. In this study, we evaluated the potential of RT-LAMP for the development of a simple and rapid detection system for FMDV RNA.

## 2. Materials and methods

### 2.1. Viral strains and samples

The strains O/CHA/1999, A/CHA/2009, C/SU/1958, Asia 1/CHA/2005 were developed RT-LAMP method and then the stain O/CHA/1999 was to test the detection limit. The serotypes O and Asia 1 were propagated in IBRS-2 cells, and the serotypes A and C were inoculated in 3-day suckling mice, respectively.

Other field isolates including classical swine fever virus (CSFV), swine vesicular disease virus (SVDV), porcine reproductive and respiratory syndrome virus (PRRSV) and Japanese encephalitis virus (JEV) were identified by RT-PCR.

A total of 139 samples included 84 FMDV, 17 CSFV, 10 SVDV, 19 PRRSV and 9 JEV specimens, which were also identified by RT-PCR (Table 1).

**Table 1:** Result comparison of RT-LAMP and RT-PCR assays using 139 samples

Pathogen	Strain (specimen number)	Results (positive number/specimen number tested)	
		
		RT-LAMP	RT-PCR
FMDV	O/CHA/1999 (N = 32)	+ (32/32)	+ (32/32)
	A/CHA/2009 (N = 22)	+ (22/22)	+ (22/22)
	Asia 1/CHA/2005 (N = 20)	+ (20/20)	+ (20/20)
	C/UN/1988 (N = 10)	+ (10/10)	+ (10/10)
CSFV	C2008 (N = 17)	-- (17/17)	-- (17/17)
SVDV	SVDV01 (N = 10)	-- (10/10)	-- (10/10)
PRRSV	HPBEDV (N = 19)	-- (19/19)	-- (19/19)
JEV	JEV2009 (N = 9)	-- (9/9)	-- (9/9)

+, positive reaction; --, negative reaction

### 2.2. RNA extraction

RNA was extracted from FMDV-infected and healthy animals, using a RNeasy Mini Kit (Qiagen) according to the manufacturer's instructions. After extraction, RNA was eluted in 60 μl of elution buffer and stored at -20°C.

### 2.3. Conventional RT-PCR and RT-LAMP

The detection of FMDV by RT-PCR was performed with primers described previously [10]. A set of four primers, F, B, FIP and BIP were designed by targeting conserved regions of FMDV 2B (Table 2). 2B nucleotide sequences of all the FMDV serotypes were retrieved from GenBank and aligned using the software program DNAStar (DNASTAR, Inc. Madison). The accession numbers used for the alignment were the following: AY593782 (A), AY593801 (A), AY593768 (A), AY593795 (Asia 1), EF149010 (Asia 1), AY593796 (Asia 1), AY593799 (Asia 1), AY593810 (C), AY593806 (C), AY593809 (C), AJ539138 (O), AY593817 (O), AF511039 (O), AY593845 (SAT 1), AY593839 (SAT 1), AY593844 (SAT 1), AY593847 (SAT 2), AY593848 (SAT 2), AY593849 (SAT 2), AY593850 (SAT 3), AY593852 (SAT 3), AY593853 (SAT 3).

**Table 2:** Details of RT-LAMP primers designed for detection of 2B coding sequences of FMDV

Primer name	Sequence
F	5'-CCTGTCGTGCATGGCCGCTGT-3'
B	5'-GAAACACGAGGCAACTTTGAC-3'
FIP	5'-CTTACAGACGAAGGTGCTGTC + CATCATGCTGGCCGACACCG-3'
BIP	5'-AGATCTCCGACTCGCTCTCCA + ACAGGACCGGTGCTCCGAAAC-3'

RT-LAMP reaction was carried out in a conventional water bath by mixing 2.0 μM each of FIP and BIP primer, 0.2 μM each of F and B primer, 1.0 mM each deoxynucleoside triphosphate, 8 U of Bst DNA polymerase (New England Biolabs) and 1 U of the THERMO-X reverse transcriptase (Invitrogen) using the manufacturer's supplied 10× buffer (containing 4 mM of MgSO_4_, 0.8 M betaine) and 1 μl of extracted template RNA in a 0.2 ml Eppendorf tube. The amplification was performed at 64°C for 45 min and then terminated by heating at 80°C for 10 min. RT-LAMP products were analyzed by 2.5% agarose gel electrophoresis.

### 2.4. Sensitivity and specificity of RT-LAMP for FMDV

Compared to RT-PCR, the detection limit of RT-LAMP was tested using the same templates at identical concentrations, which was performed in triplicate at each concentration of templates. The O/CHA/1999 RNA was quantitated using UV spectrophotometry (UNICAM 3000, US). Serial dilutions of 1, 10, 10^2^, 10^3 ^and 10^4 ^copies per reaction from FMDV strains were used in the assay. To assess the specificity of RT-LAMP, cross reactions with RNA of the strains CSFV, SVDV, PRRSV and JEV were examined. Viral RNA of the strains O/CHA/1999, A/CHA/2009, C/SU/1958 and Asia 1/CHA/2005 was used as the positive control and RNA extracted from healthy swine tissues was used as the negative control. In addition, RNA from 139 samples was extracted and subjected to FMDV RT-LAMP.

## 3. Results

### 3.1. Analysis sensitivity of the LAMP method compared to PCR. Detection limit of FMDV RT-LAMP

**Figure 1:**
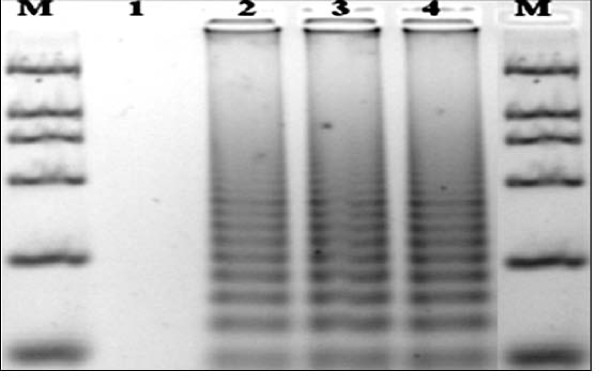
**Agarose gel electrophoresis analysis of RT-LAMP products using FMDV reference strains**. Lane M, DNA Marker DL2000 (2000, 1000, 750, 500, 250, 100 bp); Lane 1, PRRSV; Lane 2, O/CHA/1999; Lane 3, A/CHA/2009; Lane 4, Asia 1/CHA/2005.

The amplification by FMDV RT-LAMP showed a ladder-like pattern (Figure 1). The result indicated that the detection limit of FMDV RT-LAMP was 10 copies whereas that of RT-PCR was 100 copies per reaction. The detection sensitivity of FMDV RT-LAMP was therefore greater than that of RT-PCR.

### 3.2. Analytical cross reaction of FMDV RT-LAMP

RNA extracted from tissues of healthy animals and pigs infected with CSFV, SVDV, PRRSV and JEV was used as templates for RT-LAMP. Agarose gel electrophoresis analysis indicated that FMDV RT-LAMP reaction did not detect CSFV, SVDV, PRRSV and JEV as well as gave a negative reaction with tissues of healthy swine, but FMDV RNA was the positive reaction.

### 3.3. Evaluation of FMDV RT-LAMP with samples

To evaluate specificity and sensitivity of FMDV RT-LAMP, the assay correctly detected 84 FMDV positive samples but not 65 FMDV negative specimens (Table 1).

## 4. Discussion

For countries to remain FMD-free or to control FMD, rapid and accurate detection of FMDV was played a critical role in the implementation of effective countermeasures to control spread of FMD. RT-LAMP was a sensitive diagnostic method, which was quite simple, requiring only a conventional water bath or heat block for incubation under isothermal conditions [7-9]. Another useful feature of RT-LAMP is that its products can be observed directly by naked eye, because a white precipitate of magnesium pyrophosphate forms in the reaction tube [11]. Adding SYBR Green I to RT-LAMP reactions can also increase the ease detection by the naked eye [12].

Unlike RT-PCR, the greater sensitivity and specificity of RT-LAMP were reported to utilize to detect animal viruses [7,8]. The lack of cross reaction observed with CSFV, SVDV, PRRSV and host-derived RNA indicated that FMDV RT-LAMP assay was specific in addition to high sensitivity. In addition, the serotypes SAT1, SAT2 and SAT3 were not investigated in the present study due to absence of those strains or the positive clinical samples in our reference laboratory. Nevertheless, 2B was a highly conserved region in all FMDV serotypes [1], and a set of four primers targeting 2B sequence of all serotypes of FMDV were theoretically designed and likely detected seven serotypes of the FMDV infection. However, this speculation will be further indicated in the future.

RT-LAMP was a simple and timesaving procedure, allowing results to be obtained within 1 hour, whereas RT-PCR method typically requires 2 to 4 hours. Compared to RT-PCR, RT-LAMP method was a sensitive tool for the clinical diagnosis of FMDV infection. Nonetheless, the reliability of this assay should be further evaluated by large-scale investigation.

## Competing interests

The authors declare that they have no competing interests.

## Authors' contributions

HTC and JZ designed the research, and carried out most of the experiments. XTL supported experiments. HTC and YSL wrote and revised the manuscript. All of the authors approved the final version of the manuscript.
